# Morphology and histology of the olfactory organ of two African lungfishes, *Protopterus amphibius* and *P. dolloi* (Lepidosirenidae, Dipnoi)

**DOI:** 10.1186/s42649-021-00054-x

**Published:** 2021-04-17

**Authors:** Hyun Tae Kim, Jong Young Park

**Affiliations:** Department of Biological Science and Institute for Biodiversity Research, College of Natural Sciences, Jeonbuk National University, Jeonju, 54896 South Korea

**Keywords:** Aerial exposure, Evolutionary evidence, Lungfish, Mucous cell, Vomero-like epithelial crypt

## Abstract

The olfactory organs of two African lungfishes, *Protopterus amphibius* and *P. dolloi*, were investigated using a stereo microscope and a compound light microscope and were described anatomically, histologically, and histochemically. Like other lungfishes, these species present the following general features: i) elongated olfactory chamber (OC), ii) anterior nostril at the ventral tip of the upper lip, iii) posterior nostril on the palate of the oral cavity, iv) lamellae with multiple cell types such as olfactory receptor neurons, supporting cells, basal cells, lymphatic cells, and mucous cells (MC), and vi) vomero-like epithelial crypt (VEC) made of glandular epithelium (GE) and crypt sensory epithelium. Some of these features exhibit differences between species: MCs are abundant in both the lamellar and inner walls of the OC in *P. amphibius* but occur only in lamellae in *P. dolloi*. On the other hand, some between feature differences are consistent across species: the GE of both *P. amphibius* and *P. dolloi* is strongly positive for Alcian blue (pH 2.5)-periodic acid Schiff (deep violet coloration), and positive with hematoxylin and eosin and with Masson’s trichrome (reddish-brown staining), unlike the MCs of the two species which stain dark red with both Alcian blue (pH 2.5)-periodic acid Schiff and Masson’s trichrome but respond faintly to hematoxylin and eosin. The differing abundance of MCs in the two lungfishes might reflect different degrees in aerial exposure of the olfactory organ, while the neutral and acid mucopolysaccharide-containing VEC, as indicated by staining properties of the MCs, is evolutionary evidence that *P. amphibius* and *P. dolloi* are the closest living relatives to tetrapods, at least in the order Dipnoi.

## Introduction

In vertebrates, olfaction provokes ecologically fundamental behaviors (predator avoidance, migration, reproduction, food searching, etc.). The physical response is initially stimulated by the olfactory epithelium, which is in direct contact with external factors (Ache and Young 2005). The signal transduction process generally involves the following steps: begins in olfactory receptor neurons (ORNs) as a chemical stimulus above a threshold, passes along axons, and finally terminates within the olfactory bulb (OB) (Hara 1986). However, fish and higher tetrapods have different olfactory systems and electro-physiological signal pathways. Chondrichthyes and Osteichthyes have only a single main olfactory organ (MOO) with variable numbers of lamellae (Yamamoto 1982; Ferrando et al. 2016). Consequently, they use only a fluid-filled MOO that is traced to the main olfactory bulb (MOB). Contrary to lower vertebrates, tetrapods utilize not only air-filled MOOs, but also a fluid-filled vomeronasal organ that has long sensory microvilli and terminates within the accessory olfactory bulb (AOB) (Barber and Raisman 1974; Jones and Reed 1989; Halpern et al. 1995; Trotier and Døving 1998; Nowack and Wöhrmann-Repenning 2009). Interestingly, lungfish, the closest living relative to tetrapods (Brinkmann et al. 2004), have the vomeronasal organ within the olfactory organ. The lungfish vomeronasal organ has been described as a “recess epithelium” by Nakamuta et al. (2013) and a “vomeronasal-like epithelial crypt (VEC)” by Wittmer and Nowack (2017). This organ has been discussed as a unique olfactory-related structure unknown in other fish taxa (Bertmar 1981; Eisthen 1992; González et al. 2010).

The lungfish is a so-called “living fossil” as it retains primitive characteristics including well-developed lobed fins and is excellently adapted to semi-aquatic life on extremely dry land as well as under water (Jorgensen 2010). Distributed throughout Australia, South America, and Africa (Jorgensen 2010), all six lungfish species are obligate air breathers with a lung-like structure of a vascularized swim bladder. In contrast, other amphibious fishes have diverse respiratory systems such as gills, skin, buccal cavities, and intestines (Sanchez and Glass 2001; Jaafar and Murdy 2017). Such amphibious fishes also display unique olfactory organs in the nostril, chamber, and sac as well as development and composition of the sensory region and olfactory cell contents for terrestrial adaptation to air exposure (Jaafar and Murdy 2017; Kim et al. 2019; Kim and Park 2020). Among them, the lungfish olfactory organ has fascinated many researchers who concentrate on the relationship between structure and vertebrate evolution. This aspect has been studied in the West African lungfish *Protopterus annectens* (Derivot 1984), the Australian lungfish *Neoceratodus forsteri* (Theisen 1972), the spotted lungfish *P. dolloi* (González et al., 2010), and the South American lungfish *Lepidosiren paradoxa* (Wittmer and Nowack 2017). Those studies provide understanding of how lungfishes have evolved their olfactory organ to anatomically and histologically adapted to life both on land and in the water and give insight into the phylogenetic placement of lungfish in a different lineage from most extant teleost species (Marshall et al. 2011). Among the two species studied here, however, such research has not previously been carried out on the gilled lungfish *P. amphibius,* and interspecific comparisons have not been made*.*

Although lungfishes possess some vital features for terrestrial life, they show detailed differences in adaptive mechanisms: the marbled lungfishes *P. aethiopicus* and *P. annectens* are better at reducing ammonia production than is the spotted lungfish *P. dolloi*, illustrating different adaptive responses to air exposure (Loong et al. 2005). This study focuses on the anatomy and histology of the olfactory organ of two lungfishes, *P. amphibius* and *P. dolloi,* with interspecific comparison and discussion of differences from other amphibious fishes.

## Materials and methods

### Specimen preparation

**Fig. 1:**
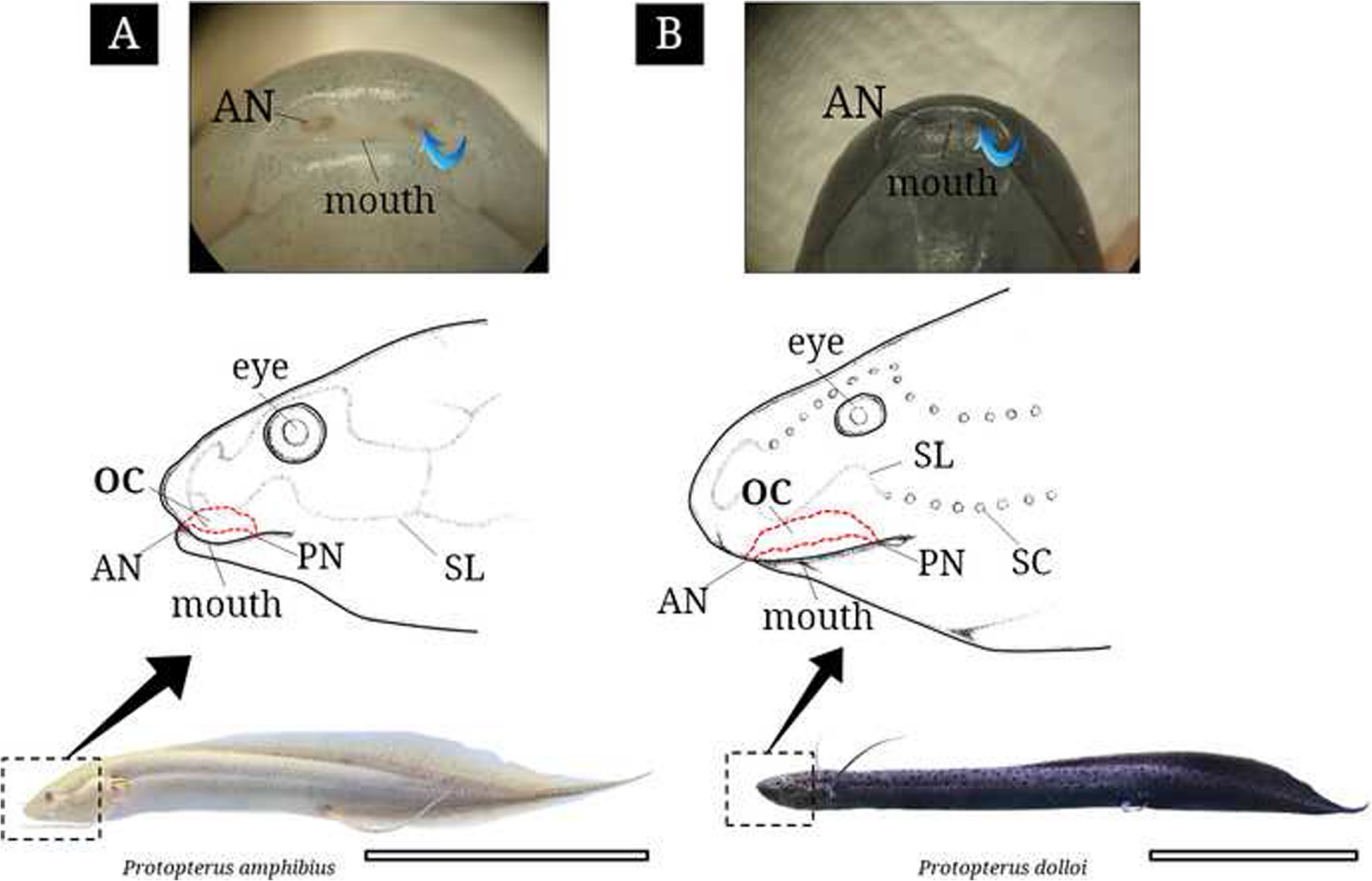
External morphology of the olfactory organ in two lungfishes, *Protopterus amphibius* and *P. dolloi*. Blue bent lines indicate incurrent water flowing. Red broken lines indicate the olfactory chamber. AN, anterior nostril; OC, olfactory chamber; PN, posterior nostril; SC, sensory canal; SL, sensory line. The bars indicate 8 cm

African lungfishes (standard length 15.5 cm and 22.2 cm in *P. amphibius*; 24.0 cm and 27.2 cm in *P. dolloi*; Fig. 1a and b) were purchased at a fish market (Aquapro), having been imported from Kenya on 26 August 2020 and from Congo on 15 September 2020, respectively. The two species are easily classified by differences in body color (uniform blue body and pale grey belly in *P. amphibius* vs. dark brown body in *P. dolloi*, Jorgensen 2010). For histology, living lungfishes were anesthetized with MS-222 (tricaine methanesulfonate; Sigma, St. Louis, MO, USA) and immediately fixed with neutral 10% formalin solution buffered at pH 7.4. All research procedures were conducted by the rules of the Jeonbuk National University Institutional Animal Care and Use Committee.

### Microscopic investigation

The fixed olfactory organs were extracted from the fishes’ snouts using a surgery blade under a dissecting scope (Stemi DV4, Carl Zeiss, Jena, Germany). The tissues were dehydrated with an ascending alcohol series (70%, 80%, 90%, 95%, 100%), cleared with xylene, and embedded in paraffin wax. The tissue-containing wax blocks were cut into five-micrometer sections; stained with either Harris’ hematoxylin and eosin (H&E), Masson’s trichrome, or Alcian blue (pH 2.5)-periodic acid Schiff (AB-PAS); and photographed under a light microscope (Imager A1, Carl Zeiss). The olfactory cells were identified with reference to Wittmer and Nowack (2017).

## Results

### External morphology

The two African lungfishes possess paired olfactory organs that are embedded at the ventral snout close to the oral cavity (Fig. 1a and b). They have a singular elongated olfactory chamber (OC) that is anteriorly open to the outside via the anterior nostril (AN) and posteriorly penetrates the palate of the oral cavity via the posterior nostril (PN). The OC contains at least 20 lamellae.

### Histology

**Fig. 2:**
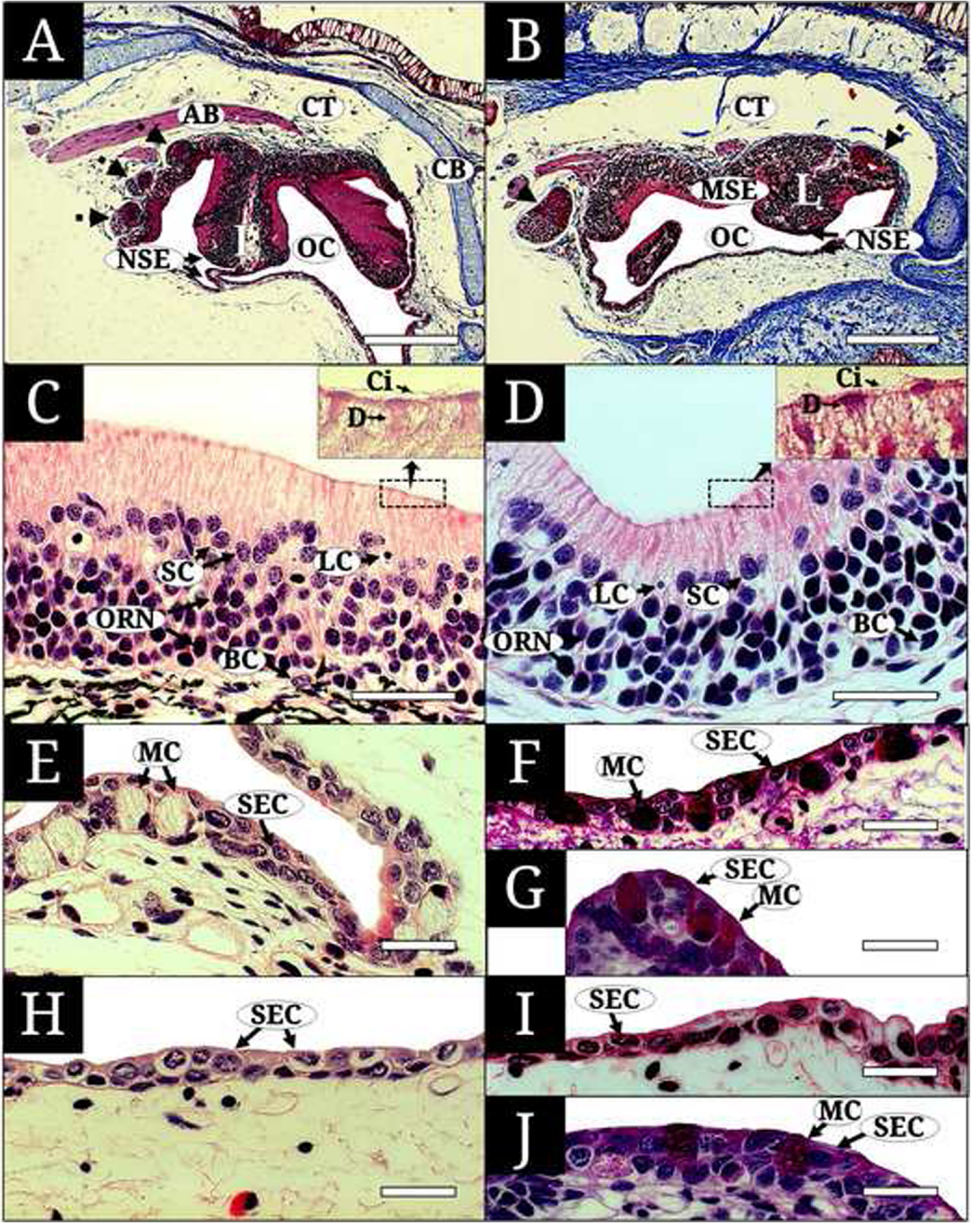
Histological characteristics of the cross-sectioned main olfactory organ in two lungfishes, *Protopterus amphibius* and *P. dolloi*, stained with hematoxylin and eosin (**c**, **d, e, h**), alcian blue (pH 2.5)- periodic acid Schiff (**f, g, i, j**), and Masson’s trichrome (**a, b**). **a**, the main and accessory olfactory organs of *P. amphibius*; **b**, the main and accessory olfactory organs of *P. dolloi*; **c**, the sensory epithelium of the main olfactory organ of *P. amphibius*; **d**, the sensory epithelium of the main olfactory organ of *P. dolloi*; **e, f**, the lateral non-sensory wall of the olfactory chamber of *P. amphibius*; **g**, the non-sensory epithelium of lamella of *P. amphibius*; **h, i**, the lateral non-sensory wall of the olfactory chamber of *P. dolloi*; **j**, the non-sensory epithelium of lamella of *P. dolloi*. BC, basal cell; Ci, motile cilia; D, dendrite; LC, lymphatic cell; MC, mucous cell; ORN, olfactory receptor neuron; SC, supporting cell; SEC, stratified epithelial cell. The bars indicate 500 μm in (**a** and **b**), 50 μm in (**c**-**j**)

In images of cross-sectioned histological material, the two lungfishes are characterized by two olfactory systems, a main olfactory organ (MOO) and a vomero-like epithelial crypt (VEC), which are separated by connective tissue and surrounded by aniline blue-positive cartilaginous bone (Fig. 2a and b). The MOO has longitudinally arranged lamellae that extend into the roof and the bottom of the OC. The lamellae consist of a sensory epithelium (SE) and a non-sensory epithelium (NSE) (Fig. 2a and b). The SE is a pseudostratified columnar layer consisting of olfactory receptor neurons (ORNs), supporting cells (SCs), basal cells (BCs), and lymphatic cells (LCs) (Fig. 2c and d). The ORNs comprise bipolar neurons with a spherical nucleus, dendrites with cilia on their apical surfaces, and an axon running into the basement membrane. Their nuclei are positive for hematoxylin, staining a dark violet color. Their dendrites and axons stain a weak pink color for acidic eosin Y staining. The cylindrical SCs have a nucleus that stains a weaker violet color than that of the ORNs and have long motile cilia on their apical surface. The oval BCs remain horizontal along the basement membrane. The LCs are very small cells with a spherical nucleus and are scattered between the SCs of the upper epithelial layer. The NSE is a stratified squamous epithelium consisting of one to three cell layers and containing stratified epithelial cells (SECs) and mucous cells (MCs) (Fig. 2e-j). The SECs make up the majority of the NSE and have a polygonal nucleus and cytoplasm that stains weakly with eosin. The MCs are large cells with a basal nucleus, numerous granules, and a faint response to H&E stain. They also stain dark red in both AB-PAS and Masson’s trichrome. Such MCs are abundant in the epithelia of the inner cells of the OC in *P. amphibius* but are lacking in *P. dolloi*.

**Fig. 3:**
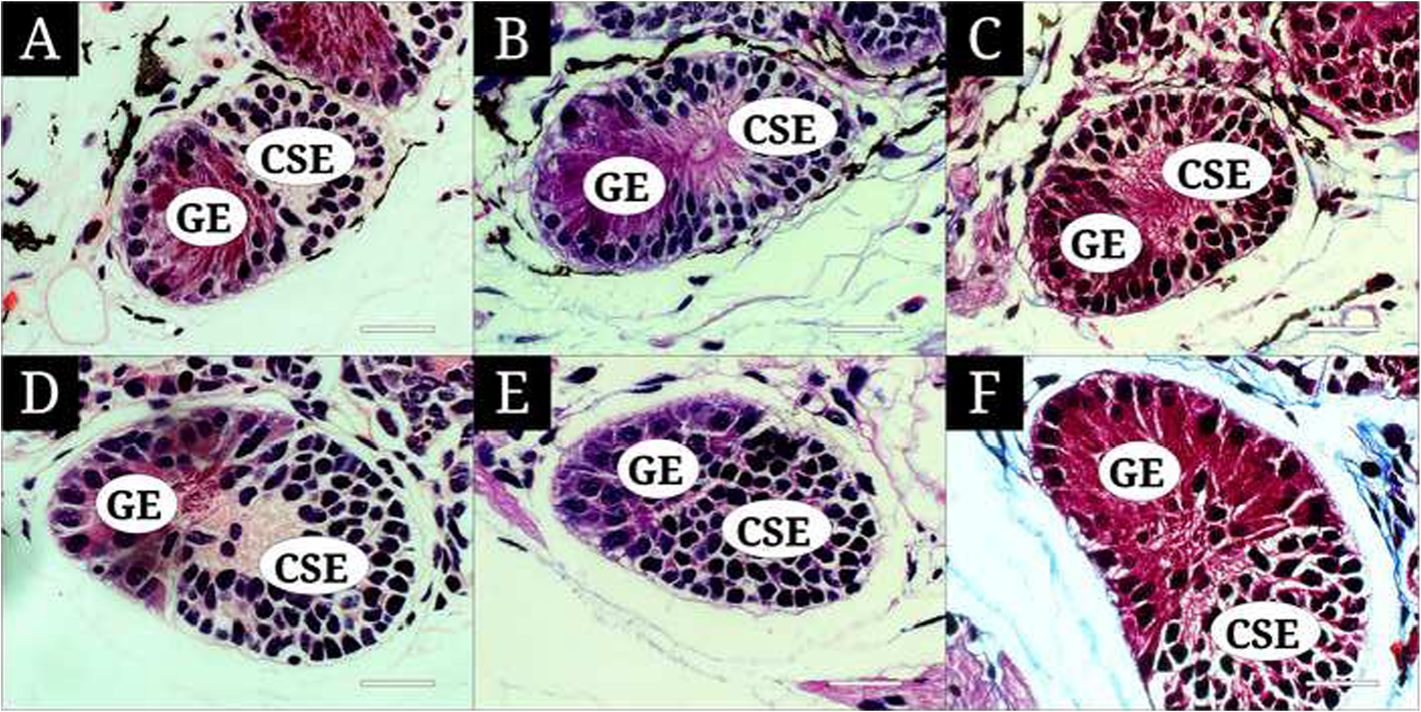
The vomero-like epithelial crypt of *Protopterus amphibius* and *P. dolloi*, stained with hematoxylin and eosin (**a, d**), alcian blue (pH 2.5)- periodic acid Schiff (**b, e**), and Masson’s trichrome (**c, f**). **a, b, c** indicate *P. amphibius* and **d, e, f** indicate *P. dolloi*. CSE sensory epithelium; GE, glandular epithelium. The bars indicate 50 μm

The VEC of both lungfishes is located under the MOO (Fig. 2a and b) and has two distinct parts, a glandular epithelium (GE) and a crypt sensory epithelium (CSE) (Fig. 3a-f). Commonly, the GE is a single layer of elongated secretory cells that have a small oval nucleus at the bottom and a slender cytoplasm extending from the basal to the central region of the VEC. With staining, the GE stains a reddish-brown color with H&E and Masson’s trichrome and a dark violet color with AB-PAS. The CSE is a pseudostratified epithelium with bipolar neurons, presenting nuclei in diverse layers of the epithelium. It stains a weak pink with H&E, violet with AB-PAS, and brown with Masson’s trichrome.

## Discussion

The olfactory organs of two extant lungfish, *Protopterus amphibius* and *P. dolloi*, which retain primitive characteristics and have the ability to breathe air, were investigated and compared to those of other amphibious fishes that possess excellent tolerance to being out of water. Based on morphological and histological study, the olfactory organs of the two lungfish species revealed the following primitive characteristics: i) an elongated OC with several lamellae, ii) an AN at the ventral tip of the upper lip, iii) a PN at the palate of the oral cavity, iv) a MOO with longitudinal lamellae, v) lamellae consisting of ORNs, SCs, BCs, LCs, and MCs, and vi) a VEC made up of the GE and the CSE, as reported in other lungfishes (Derivot 1984; Nakamuta et al. 2013; Wittmer and Nowack 2017). Among these features, the elongated lungfish OC resembles those of amphibious mudskippers of the subfamily Oxudercinae (Kuciel et al. 2013). In particular, the Asian swamp eel *Monopterus albus* possesses a pipe-like chamber penetrating the dorsal snout (Kim 2018). Nevertheless, there are some differences in the organ structure between lungfishes and other amphibious fishes. First, the two lungfishes have several folded lamellae ascending from the inner wall of the OC, whereas mudskippers have no such suspended lamellae (Kuciel et al. 2013; Kim et al. 2019). In other lungfish, the number of lamellae per one rosette row was 8 to 37 in adult *P. annectens* (80–550 mm in total body length) and 2 to 26 in adult *Lepidosiren paradoxa* (80–560 mm) (Nakamuta et al. 2013; Wittmer and Nowack 2017). Unfortunately, we could not obtain useful statistical data on the lamellae due to a shortage of specimens (only two lungfish per species). Nevertheless, we confirmed that that *P. amphibius* and *P. dolloi* have at least 20 lamellae each. In this respect, although mudskippers and lungfishes both successfully live a terrestrial life with aerial exposure, the dependance on the sensory organ might be different between two. More advanced mudskippers commonly have remarkably large eyes as an adaption to terrestrial activity (Sayer 2005; Jaafar and Murdy 2017), whereas lungfishes, which estivate in a cocoon when exposed to air, possess unremarkable “degenerate” eyes (Marshall et al. 2011).

Second, the two lungfishes possess no accessory nasal sac or any appendage for olfactory ventilation, unlike mudskippers that have one or two sacs (Kuciel et al. 2013). In amphibious fish, the accessory nasal sac generates a suction force toward the OC by increasing the internal pressure and helps take up water in shallow stagnant water or on wet land with little water (Nevitt 1991; Kuciel 2013). Unlike mudskippers, the OC of the two lungfishes has no ANS but penetrates toward the oral cavity. We hypothesize that the much larger oral-cavity volume of *P. amphibius* and *P. dolloi*, relative to ANS-containing amphibious fishes, is used for odorant residue elimination.

Of particularly interest is the distribution of MCs in the two lungfish species studied here: MCs are abundant in both the lamellae and chamber inner wall in *P. amphibius* but are scarce and only found in the lamella in *P. dolloi*. In fish skin, the mucus secreted by MCs has a large range of physiological functions: i) protection against harmful chemical or physical factors, ii) disease resistance, iii) ion and water regulation, and iv) respiratory gas exchange (Shephard, 1994). In addition, Horn and Riegle (1981) and Laming et al. (1982) found that enhanced mucous secretions prevent water loss in stichaeoid fish and shanny (*L. pholis*) exposed to aerial conditions. In this view, *P. amphibius*, with more abundant MCs than *P. dolloi*, can be regarded as having a different cytological strategy for aerial exposure of the olfactory organ.

Histochemically, the properties of the MCs in teleost olfactory organ can be classified into acidic or neutral mucopolysaccharide and glycoprotein according to the mixed ratio of protein and carboxylate. As the two lungfishes show a deep red color with AB (pH 2.5)-PAS but a faint color with H&E, it was concluded that MCs have only a neutral mucopolysaccharide with less ionized amino acids (Gona 1979).

The GE of the VEC in *P. amphibius* and *P. dolloi* was different from the MCs of the MOO: reddish-brown vs. faint with H&E and Masson’s trichrome and dark violet vs. dark red with AB (pH 2.5)-PAS. These features of the GE are due to acid and neutral mucopolysaccharide with much protein mass, as in other lungfish species with much granule protein (Nakamuta et al. 2013; Wittmer and Nowack 2017). Nevertheless, additional approaches are needed for identifying proteins.

Wittmer and Nowack (2017) reported two types of secretary glands, a glandular duct cell and an elongated glandular cell (glandular epithelium), but we confirmed only one type of secretory gland, the GE, in the VEC to produce mucus. The GE with AB (pH 2.5)-PAS reaction has been variably expressed in lungfishes: PAS positive but Alcian blue negative in *P. annectens* (Nakamuta et al. 2013), Alcian blue (pH 2.5) positive but PAS negative in *L. paradoxa*, and both Alcian blue and PAS positive in *P. annectens* (Wittmer and Nowack 2017). Tierney (2015) stated that glandular secretion of proteins is not important in fish olfaction. Meanwhile, Wittmer and Nowack (2017) suggested that protein-containing mucopolysaccharide participates in the sensory mechanism at least in the lungfish olfactory system, and the Alcian blue-positive GE functions to remove odorant residues with acidic mucus. Based on our results, we think that the VEC is deeply related to lungfish olfaction.

Consequently, we confirmed that the difference in density of MCs in *P. amphibius* and *P. dolloi* reflects different degrees of aerial exposure during their lives, and acid and neutral mucopolysaccharide-containing VEC is evolutionary evidence for lungfishes as the closest living relative to tetrapods, at least in the order Dipnoi.

## Conclusion

Two African lungfishes, *Protopterus amphibius* and *P. dolloi*, are the closest living relatives to tetrapods and are obligate air breathers excellently adapted to a semi-aquatic lifestyle that exploits dry land and freshwater environments. The elongated olfactory chamber; the anterior nostril at the ventral tip of the upper lip; the posterior nostril penetrating the oral palate; the main olfactory organ with several lamellae; the lamella consisting of olfactory receptor neurons, supporting cells, basal cells, lymphatic cells, and mucous cells; and the vomero-like epithelial crypt are general characteristics shared with other lungfishes. Among them, MC density are unique between the two lungfishes and the VEC of *P. amphibius* and *P. dolloi* is very similar in mucopolysaccharide and protein composition. The different MC densities between these two African lungfishes are related to different degrees of aerial exposure of the olfactory organ, and the VEC is evolutionary evidence for lungfishes as the closest living relative to tetrapods, at least in the order Dipnoi.

## Data Availability

Not applicable.
